# Comparison of misfit and roughness of CAD-CAM ZrO, selective laser sintered CoCr and preformed Ti implant abutment crowns

**DOI:** 10.1186/s12903-024-04735-3

**Published:** 2024-08-25

**Authors:** Fahim Vohra, Rawan Alsaif, Rawaiz Khan, Ishfaq A Bukhari

**Affiliations:** 1 Department of Prosthetic Dental Sciences, College of Dentistry, King Saud University, Kingdom of Saudia Arabia, Riyadh, 12485 - 6541 Saudi Arabia; 2 Department of Research, College of Dentistry, King Saud University, Kingdom of Saudia Arabia, Riyadh, 12485 - 6541 Saudi Arabia; 3 Department of Pharmacology, College Of Medicine, King Saud University, Kingdom of Saudia Arabia, Riyadh, 12485 - 6541 Saudi Arabia

**Keywords:** Selective laser sintering, Implant, Abutment, CAD-CAM, Zirconium oxide

## Abstract

**Background:**

Marginal misfit and surface roughness of customized implant abutments is critical for restorative success. However, little is known about the comparison of misfit and surface roughness of CAD-CAM Zirconium oxide (ZrO), selective laser melting (SLM) Cobalt Chrome (CoCr) and preformed abutments. The aim of the study is to investigate the relation of misfit and micro-roughness of selective laser melting (SLM), preformed and CAD-CAM implant abutments.

**Methods:**

Thirty internal connection, endosseous dental implants (Ø 4.0 mm x 10 mm, Dentium) were mounted in Polymethyl methacrylate vertically. Ten preformed Titanium alloy (Ti) abutments with 1 mm soft tissue height and Ø 4.5 mm were included as controls. Ten each of Y-TZP and SLM-CoCr, abutment/crowns were fabricated using CAD-CAM milling (CAD-CAM-ZrO) and SLM techniques. Surface micro-roughness (Ra) of the fabricated implant abutment/crown was evaluated with a 3D optical non-contact microscope. All implant restorations were torqued to implants (30 Ncm) using a Tohnichi BTGE digital torque gauge and were analyzed with Bruker micro-CT (Skyscan 1173) to detect micro-gaps at pre-selected points at implant abutment interface. The Ra and misfit data were compared using ANOVA, Tukey-Kramer, Kruskal-Wallis test and Pearson correlation (*p* < 0.05).

**Results:**

Mean Ra among SLM CoCr abutments [0.88 (0.09) µm] were lower than CAD-CAM-ZrO and higher than preformed Ti abutments. Horizontal misfit among SLM-CoCr [45.43 (9.41) µm] and preformed Ti [36.87 (13.23) µm] abutments was not statistically different (*p* > 0.05). Misfit was significantly higher in Y-TZP samples compared to SLM-CoCr (*p* = 0.031) and preformed Ti abutments (*p* = 0.01). Preformed Ti abutments showed significantly lower misfit compared to SLM-CoCr abutments (*p* = 0.01). A positive linear correlation was observed between the surface roughness (Ra) and vertical misfit (*r* = 0.61, *p* < 0.05).

**Conclusion:**

SLM CoCr abutments showed rough surface compared to preformed Ti abutments, while horizontal misfit was comparable among SLM-CoCr and preformed abutments. Misfit was significantly greater in Y-TZP abutments, compared to SLM and preformed abutments. SLM abutment fabrication technique needs further improvement to provide better fit and surface topography.

## Introduction

A stable implant-abutment interface (IAI) is critical for the long-term success of implant based.

oral rehabilitation [1, 2]. Among other properties, passivity of fit and absence of micro-gap at the IAI is of paramount importance for good biological and mechanical prognosis of the restoration [3–6]. The micro-gap at IAI is defined as “the microscopic space that exists between the implant body and abutment” [7]. It is impossible to achieve an IAI with no gaps, as there is lack of precision in abutment fabrication methods. Misfit or micro-gap at IAI increase the risk of microleakage and microbiological plaque accumulation during intra-oral function, increasing the potential for tissue inflammation (mucositis and peri-implantitis) [8, 9]. Furthermore, occlusal forces during function around a mis-fitting IAI are magnified resulting in mechanical failures including, prosthetic screw loosening and fractures [10].

Preformed implant abutments are commonly employed for restoring dental implants and display superior mechanical properties in comparison to cast customized abutments^11^. However, the use of customized abutments are pivotal for satisfying functional, esthetic and biological requirement. Cast and CAD-CAM implant abutments are the most common manufacturing techniques for Implant rehabilitations. Casting, although common, is technique sensitive and involves multiple indirect steps which are operator dependent, increasing the susceptibility to errors [11]. CAD-CAM on the other hand, is digitally managed and has proven to be accurate, efficient, and convenient. CAD-CAM technique has low cost effectiveness, due to loss of material after milling [12]. Therefore, there is a need for a method which can produce customized, cost-effective and accurate implant abutments and restorations for better long-term prognosis of implant restorations.

Additive manufacturing technique (AMT) is a new method for abutment fabrication, which is defined as ‘‘a process of joining materials to make objects from 3D modelled data, usually layer upon layer” [13]. Other terms for 3D printing and additive manufacturing are “rapid prototyping”, “rapid manufacturing”, “layered manufacturing”, and “freeform fabrication”. The general categories of 3D printing include, extrusion printing, inkjet printing, laser melting/ sintering and lithography printing [14]. AMT for resins, employs extrusion methods, however metal alloys are processed selective laser melting (SLM) or selective laser sintering (SLS). SLM utilizes a laser for a light source that generates a 3D structure through welding and sintering of a dispensed material at a high temperature. Sintering continues as the stage moves down and the material is added layer-by-layer that is embedded in powder bed [15]. SLM is employed in fabrication of definitive indirect restorations including implant abutments. Existing literature on misfit of SLM tooth supported crowns and frameworks have reported similar outcomes to cast and milled fabrication methods [16]. However, literature available on the use of SLM in fabrication of implant abutments is limited [17, 18].

A study compared the surface topography and misfit of milled, SLM, and cast implant abutments [17]. The concluded that the milled components had low roughness than cast or sintered abutments, with no statistically significant difference among sintered and cast restorations [17]. In a similar investigation, preformed Ti was compared to SLM CoCr abutments with lower misfit for Ti abutments [18]. The current literature does show comparison of marginal misfit of abutment, however, the relation of surface topography and internal misfit of implant abutments fabricated with CAD-CAM Zirconia, preformed Ti and SLM CoCr is insufficient [17, 18]. It is hypothesized that microroughness and abutment internal adaptation of SLM (3D-printed) implant abutments will be comparable to preformed and CAD-CAM abutments. Therefore, the aim of the study is to investigate the relation of misfit and micro-roughness of SLM, preformed and CAD-CAM implant abutments.

## Materials and methods

To identify the suitable sample size, a power analysis was conducted using data from a similar investigation [19]. With parameters of 80% power, a 95% confidence interval (α = 0.05), and 0.6 effect size, a minimum sample size of 8 specimens was calculated. Considering potential sample failures, a decision was made to include 10 samples per group.

Thirty internal connection, endosseous dental implants (Ø 4.0 mm x 10 mm, Dentium Co., Seoul, Korea) were mounted in Polymethyl methacrylate (Major OrthoTM, Torino, Italy) vertically (Ney surveyor; Dentsply-Sirona Inc., York, PA, USA) with 2 implant threads exposed. Ten preformed Titanium alloy (Ti) abutments with (Dual abutment, Dentium Co., Seoul, Korea) 1 mm soft tissue height and Ø 4.5 mm were included as controls (Preformed Ti). Ten Zirconium oxide (ZrO) abutment crowns were fabricated using CAD-CAM milling (Y-TZP-CAD-CAM, Prettau Zirconia, ZirconZahn, An der Ahr, Gais, Italy ) and ten Cobalt Chromium (CoCr) alloy abutment crowns were fabricated using selective laser melting (SLM), (SLM-CoCr).

### Specimen Fabrication

**Fig. 1:**
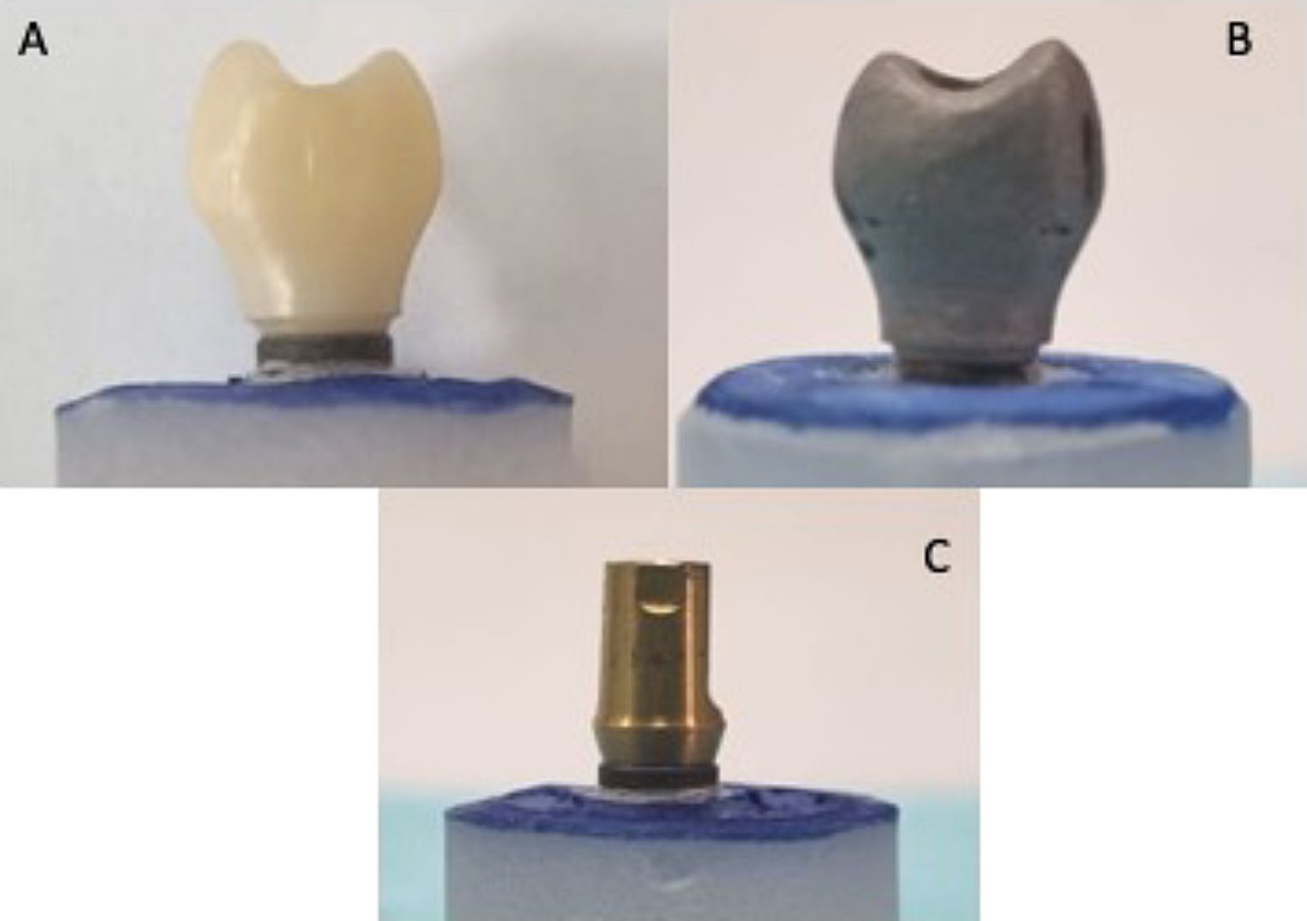
Implant abutment samples. **(A)** CAD-CAM ZrO; **(B)** SLM-CoCr; **(C)** Preformed Ti

Fabrication of ZrO abutment crowns were performed using ZirconZahn (An der Ahr, Gais, Italy) system. A scan-marker for Dentium System (ZirconZahn; An der Ahr, Gais, Italy) with 4.8 mm diameter was scanned with Zirkonzahn optical scanner (S600 ARTI, ZirconZahn; An der Ahr, Gais, Italy). The platform of the abutment (internal connection) was chosen from the Dentium superline library provided by Dentium to the technical laboratory and the coronal part of the crown was designed with standard mandibular second premolar dimensions from the library. The virtual implant abutment crown model standard tessellation language (STL) file was saved. Abutment crowns were milled employing the ZrO material blanks (Prettau Zirconia- ZirconZahn; An der Ahr, Gais, Italy- partially stabilized with yttrium and enriched with aluminium) using the milling machine (Ceramill Motion, Amann Girrbach, Herrschaftswiesen 1, Koblach, 6842 Austria). Ten SLM- CoCr abutments, crowns were fabricated with the already designed STL file. The single piece abutment model was transferred to concept laser machine (mlab cusing metal laser melting system; GE Additive company, Boston, USA) using CoCr alloy (Starbond Easy Powder 30; Scheftner GmbH, Mainz, Germany); and Sisma (mysint 100 3D laser metal fusion technology; via dell’Industria, Italy) using Ti 6Al–4 V powder grade 23 (TI64GD 23, LPW Technology Ltd, United Kingdom), with specific recommended parameters. The fabricated specimen including the ZrO-CAD-CAM, SLM CoCr and preformed implant restorations are presented in Fig. 1(A, B, C).

### Microroughness (Ra) evaluation

The surface micro-roughness (Ra) of the fabricated implant abutment and crown was evaluated with A 3D optical non-contact microscope (Contour GT-K 3D Optical Microscope, Bruker^®^,Tucson, Arizona, USA). For each abutment and crown specimen, scans were performed at five points on the hex at 5 intervals. A mean of all Ra outcomes for each specimen was identified. Calibrations were performed prior to scans and a Control and Analysis Software (Vision 64, Bruker^®^, Tucson, Arizona, USA) was employed in accordance with the manufacturer’s recommendation, to manage the accuracy of measurements of surface roughness.

### Implant abutment misfit (µm)

All implant abutment and crowns specimens were secured to their specific mounted implants with a Ti abutment screw (Dentium, Co., Seoul, Korea) with 2 mm diameter. All implant abutment and crowns were torqued to implants (30 Ncm) using a Tohnichi BTGE digital torque gauge (Tohnichi Mfg, Tokyo, Japan). The secured specimens in the specific groups (Y-TZP-CAD-CAM, SLM-CoCr and Preformed Ti) was analyzed with Bruker micro-CT (Skyscan 1173 high-energy spiral scan micro CT; Skyscan NV, Kontich, Belgium) to detect micro-gap at pre-selected points at implant abutment interface. Each mounted sample was positioned in chamber with the standard parameters. Numerical parameters needed to establish the best image results were checked and adjusted. A ring artifact reduction of 5 for non-uniformity of the background image taken by the x-ray camera; 25% beam hardening compensation to prevent the specimen from appearing artificially denser at or near its surface, and less dense at its central parts; and a smoothing of 2 using Gaussian kernel were applied. A 16-bit TIF file format was the choice selected for saving the images because of the variety of densities comprising the specimen. Using N Recon^®^ software (program version 1.6.1.3, Bruker Skyscan, Kontich, Belgium) 3D reconstruction of images were performed. The parameters for reconstruction of images, included image slice thickness of 14 μm and number of slices to be 10,890.

**Fig. 2:**
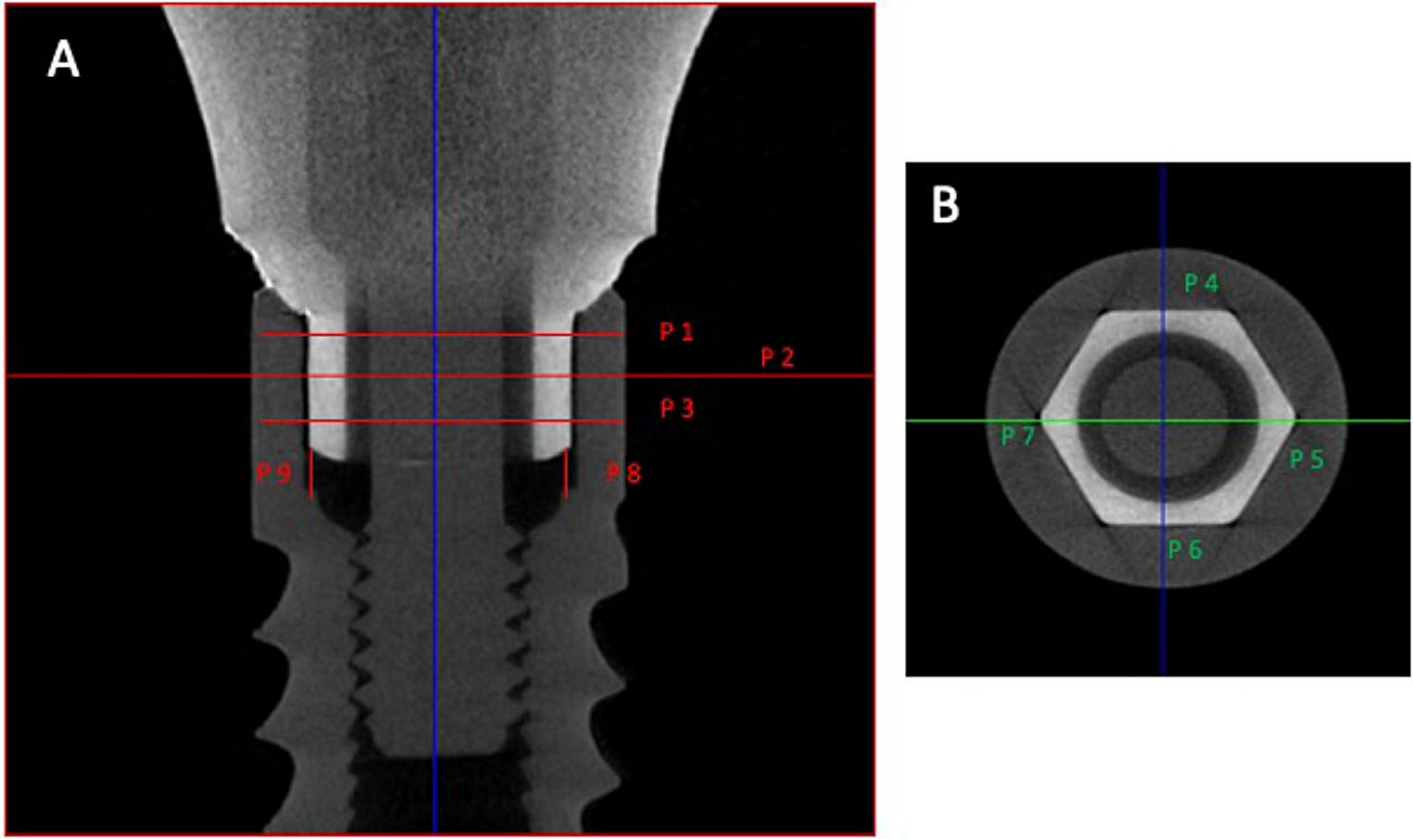
Assessment points at the implant abutment connection **(A)** Vertical cross-section **(B)** Horizontal cross-section

Reconstructed images were 3D registered and loaded in the Dataviewer^®^ Software (BrukerSkyscan, Kontich, Belgium). Each image produced by scan was analyzed with the software along with measurements for horizontal and vertical adaptation at predetermined points, as adapted from a previous study [20]. The measurements were preformed between the outer surface of the abutment and the inner surface of the implant. In the coronal cross-section, evaluations were at 3 levels for horizontal (p1, p2 & p3) misfit and four circumferential points (p4, p5, p6 & p7), resulting in twelve horizontal misfit measurements on each specimen (Fig. 2). For vertical misfit assessment, two evaluations were performed from the external-apical surface at the abutment hex excluding the bevel, to the implant shoulder (p8 & p9) (Fig. 2). All machines including the non- contact surface profilometer and the Micro- CT, were calibrated prior to the use in the patient samples.

The normality of data was assessed using Kolmogorov-Smirnov test. Means and standard deviations of Ra and misfit were compared using Analysis of variance (ANOVA) and Tukey-Kramer multiple comparisons tests. Vertical misfit was assessed using Kruskal-Wallis test (*P* < 0.05). Pearson correlation was employed to evaluate dependency between independent variable (surface roughness) and dependent variables (vertical and horizontal misfit).

## Results

**Fig. 3:**
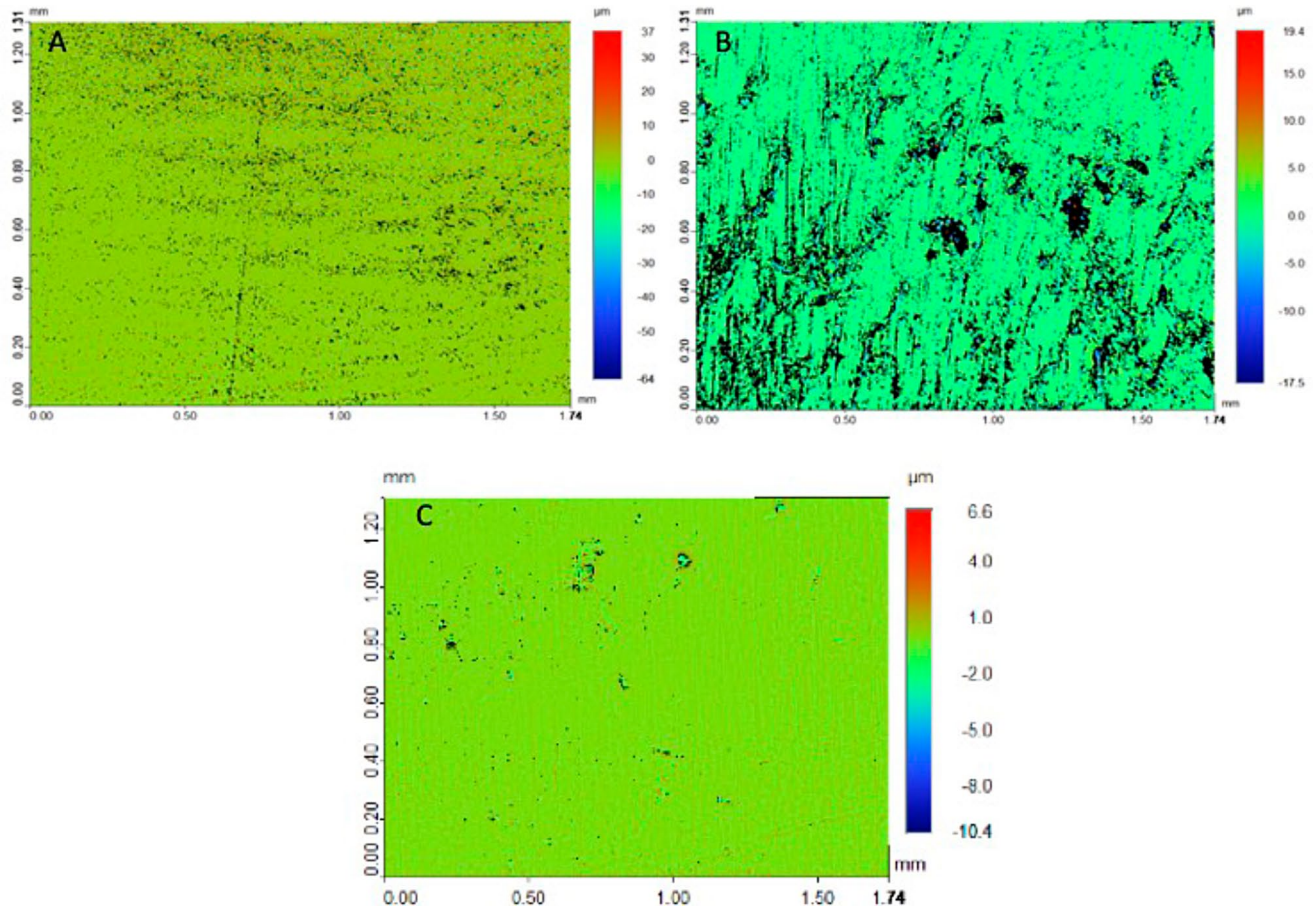
Surface roughness micrographs among the study groups. **A** CAD-CAM ZrO; **B** SLM-CoCr; C. Preformed Ti

Table 1 presents the mean and standard deviations of surface microroughness (Ra) (µm) among study groups. Preformed Ti abutment surface showed the lowest Ra [0.33 (0.11) µm], while Y-TZP CAD-CAM abutment showed the highest Ra [2.24 (0.26) µm] values. Mean Ra among SLM CoCr abutments [0.88 (0.09) µm] were lower than CAD-CAM-ZrO and higher than Preformed Ti abutments respectively. A significant difference in Ra among the study groups was observed (*p* < 0.05) (Table 1). The surface roughness among CAD-CAM-ZrO abutments was significantly higher (< 0.01) compared to SLM CoCr and Preformed Ti respectively (Table 1). Ra for SLM CoCr, was higher (*p* < 0.05) compared to Preformed Ti abutments. Micrographs of Ra among the study groups are presented in Fig. 3.

**Table 1:** Comparison of mean Ra (µm) among study groups

Abutment type	Mean (SD) Ra	Comparison groups	Mean Dif.	*P* value
CAD-CAM-ZrO	2.24 (0.26)	P-Ti	1.91 (0.15)	< 0.01
		SLM-CoCr	1.36 (0.17)	< 0.01
SLM CoCr	0.88 (0.09)	CAD-CAM-ZrO	-1.36 (0.17)	< 0.01
		P-Ti	0.55 (0.02)	< 0.05
Preformed Ti	0.33 (0.11)	CAD-CAM-ZrO	-1.91 (0.15)	< 0.01
		SLM-CoCr	-0.55 (0.02)	< 0.05

Ti. Titanium; ZrO. Zirconium oxide; Ra. Roughness; CoCr. Cobalt chromium

SLM. Selective laser melting; P. Preformed

**Fig. 4:**
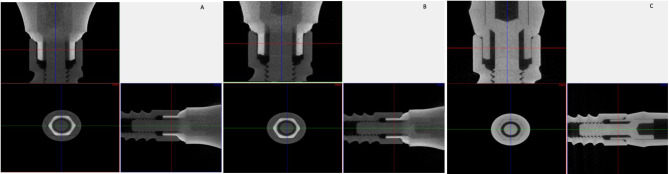
MicroCT scans for misfit assessment among the groups. **A**. CAD-CAM ZrO; **B**. SLM-CoCr; C. Preformed Ti

**Fig. 5:**
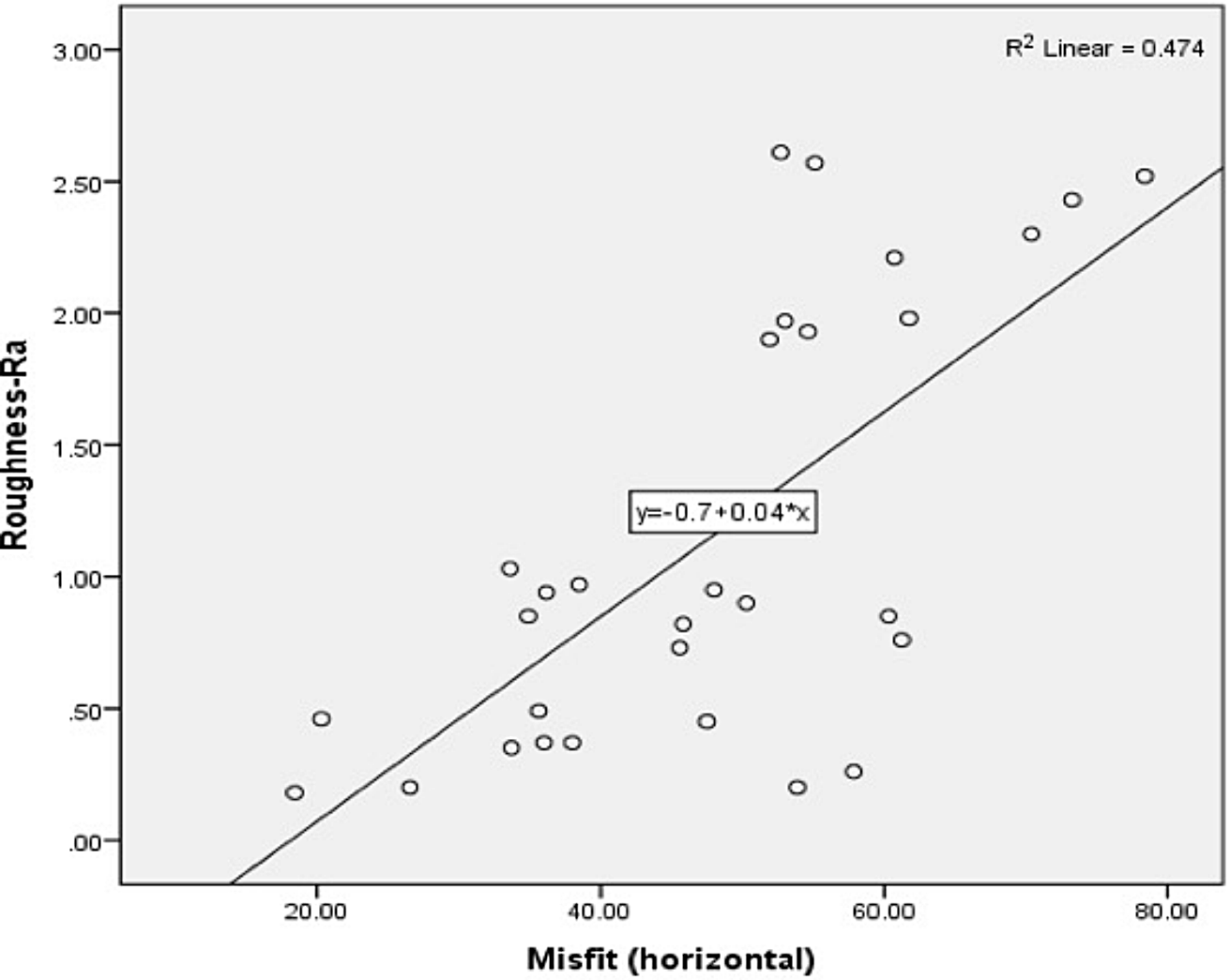
Comparing roughness Ra and horizontal misfit

The horizontal misfit (µm) among the study groups is presented in Table 2. CAD-CAM-ZrO abutments [61.19 (9.12) µm] showed highest misfit, however, lowest horizontal misfit was observed among Preformed Ti abutments [36.87 (13.23) µm]. A significant difference among the horizontal misfit of study groups was observed (*p* < 0.05). CAD-CAM-ZrO abutment showed significantly higher horizontal misfit compared to SLM CoCr (*p* = 0.02) and Preformed Ti abutments (*p* = 0.01). Horizontal misfit among SLM CoCr [45.43 (9.41) µm] and Preformed Ti [36.87 (13.23) µm] abutments was not statistically different (*p* > 0.05) (Table 2) (Fig. 4A, B and C). Correlation between the surface roughness and horizontal misfit was *r* = 0.47, with *p* < 0.05 exhibiting a positive linear correlation (Fig. 5).

**Table 2:** Means and standard deviations of horizontal misfit (µm) among study groups

Study groups	*N*	Minimum	Maximum	Mean (SD)	*p* value*
CAD-CAM-ZrO	10	52.07	70.31	61.19^a^ (9.12)	< 0.05
SLM CoCr	10	32.12	54.84	45.43^b^ (9.41)	
Preformed Ti	10	23.64	50.1	36.87^b^ (13.23)	

*ANOVA, different superscript small alphabets denote significant difference

Ti. Titanium; ZrO. Zirconium oxide; Ra. Roughness; CoCr. Cobalt chromium

**Fig. 6:**
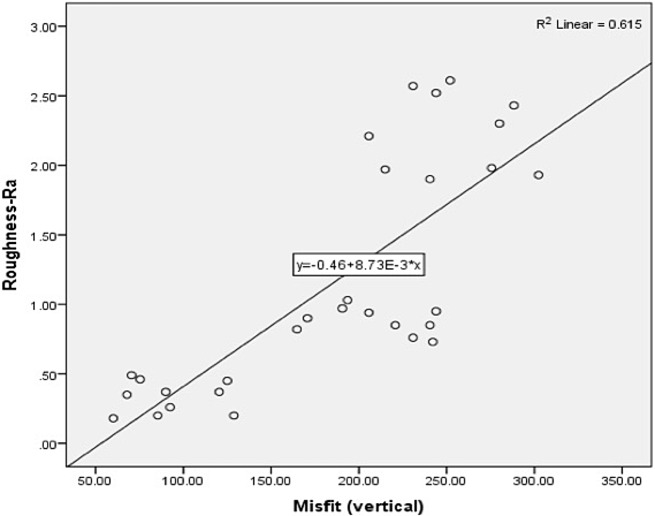
Comparing roughness Ra and vertical misfit

SLM. Selective laser melting; P. Preformed;

Table 3 presents the means and standard deviations of vertical misfit among the study groups. CAD-CAM-ZrO abutments showed higher vertical misfit [253.63 (32.20) µm], while Preformed Ti abutments [91.77 (25.01) µm] showed lowest misfit outcomes (Fig. 3). Vertical misfit among SLM CoCr samples was 210.36 (28.15) µm. A statistically significant difference for vertical misfit among the groups (*p* < 0.01) was observed. Misfit was significantly higher in ZrO samples compared to SLM CoCr (*p* = 0.031) and Preformed Ti abutments (*p* = 0.01). Preformed Ti abutments showed significantly lower misfit compared to SLM-CoCr abutments (*p* = 0.01) (Table 3). A positive linear correlation was observed between the surface roughness (Ra) and vertical misfit (*r* = 0.61, *p* < 0.05) (Fig. 6).

**Table 3:** Mean and standard deviations of vertical misfit among study groups

Study groups	*N*	Minimum	Maximum	Mean (SD)	*p* value*
CAD-CAM-ZrO	10	220.53	285.83	253.63 ^a^ (32.20)	< 0.01
SLM CoCr	10	181.21	239.71	210.36 ^b^ (28.15)	
Preformed Ti	10	66.76	116.80	91.77 ^c^ (25.01)	

*ANOVA, different superscript small alphabets denote significant difference. Ti. Titanium; ZrO. Zirconium oxide; Ra. Roughness; CoCr. Cobalt chromium

SLM. Selective laser melting; P. Preformed

## Discussion

The present study was based on the hypothesis that micro-roughness and internal adaptation of SLM (3D-printed) implant abutments will be comparable to preformed and CAD-CAM abutments. SLM abutments exhibited low roughness than CAD-CAM abutments, but higher roughness compared to preformed Ti abutments. In addition, SLM abutments showed higher misfit than preformed and lower misfit compared to CAD-CAM abutments. Therefore, the hypothesis was rejected. Multiple reasons are implicated for the outcomes of the present study including, material type, assessment technique, abutment geometry and limitations of fabrication techniques.

The Ra values observed in the present study were well above the recommended values (0.2 μm), as no surface finishing was performed, to assess the influence of SLM, CAD-CAM and machining techniques [21]. Ra for preformed abutments [0.33 (0.11) µm] reflects the effect of the machining process. However, it was higher than the Ra observed for preformed abutments in previous studies [22, 23]. The CAD-CAM ZrO abutments showed higher Ra than SLM abutments due to the polycrystalline diamond structure of ZrO and milling parameters. Factors including material removal rate, higher feed per tooth teeth, surface abrasion due to diamond cutting and tool wear are possible contributing factors in the increased Ra for the CAD-CAM ZrO abutments [24]. By contrast, a previous study by Fernandez et al., [17] showed similar surface roughness for SLM and cast abutments, however comparison of CAD-CAM ZrO to SLM CoCr abutment surface roughness is not available.

In the present study, SLM CoCr showed higher Ra compared to preformed abutments. It is suggested that factors including, multiple deposition layers (staircase effect), present of partially melted particles, porosity formation during sintering and un-melted areas during the SLM fabrication technique contributes to roughness [25]. In a recent study, the influence of particle size in SLM method for printing metal restorations was highlighted, with the suggestion of utilizing finer alloy powder to minimize roughness [26]. Nagarajan et al., in a recent study recommended the use of finer powder to minimize surface roughness [25]. Interestingly, presence of incompatible alloy particle size to laser spot size or layer thickness influences the melt pool behavior and surface topography of the printed metal [25].

The horizontal and vertical misfit at the IAI were assessed using micro-computed tomography (micro-CT) to detect the micro-gap in 3 dimensions as a non-destructive method. Previous studies have evaluated the IAI misfit, however methodology and location points were inconsistent [17, 18]. In a study by Son et al., five evaluation methods were used to evaluate the marginal and internal fit of indirect castings [27]. In the present study, the 3D images of abutments were oriented according to the first thread of the implants; which was similar in all samples. In addition, the position of points for measurements of misfit were also related to implant threads, making the evaluations repeatable.

In the present study, SLM abutments showed higher horizontal misfit compared to preformed abutments. It is suggested that the high surface micro-roughness of SLM group produced increase in the horizontal micro-gap, as positive correlation was observed between roughness and misfit. The preformed abutments showed significant lower vertical misfit among the groups. These results are.

in agreement with other studies, which showed comparable vertical misfit between the sintered CoCr and cast CoCr abutments [17]. Furthermore, in a similar study, sintered CoCr abutments exhibited significant higher vertical misfit than preformed abutments [18]. It is suggested that increasing the magnitude of applied torque to abutment screws improves the compression at the implant abutment interface, increasing the stability at the joint [28]. Various implant manufacturing companies recommend abutment screw torque values from 25 to 40 Ncm. In the present study the abutment screwtorque employed was 30Ncm, however, it remains to be identified if increasing the torque in the study setup would decrease the horizontal and vertical misfit values. Although the study showed critical outcomes regarding misfit and surface roughness of SLM implant abutments, the comparable outcomes among different abutment types may differ in intra-oral conditions. In addition, the customized abutments were not subjected to finishing procedures; this could have possibly affected the study roughness outcomes. Previous studies have shown that implant abutment surface treatments including sandblasting, acid etching, UV light have shown significant influence on the surface roughness [29] and possibly influence the misfit at the abutment to implant interface. However, further studies are recommended in this regard. The outcomes of vertical misfit in the present study showed variations and wide standard deviations among groups. This is due to the internal design of the preformed and customized abutments.

## Conclusion

Within the limitation of the present study the following can be concluded, that SLM CoCr abutments showed rough surface compared to preformed Ti abutments, while ZrO CAD-CAM showed highest roughness. ZrO CAD CAM showed highest misfit, while SLM CoCr and preformed abutments showed similar misfit outcomes. SLM abutment fabrication technique needs further improvement to provide better fit and surface topography, however randomized controlled trials are recommended to evaluate their clinical impact.

## Data Availability

All data generated or analyzed during this study are included in this published article.
